# Occupational future time perspective and employability: the role of decent work perceptions in Chinese college students

**DOI:** 10.3389/fpsyg.2025.1712125

**Published:** 2026-01-23

**Authors:** Hongxia Ming

**Affiliations:** College of Physics and Electronic Information, Dezhou University, Dezhou, China

**Keywords:** Chinese college students, decent work perceptions, employability, focus on limitations, focus on opportunities, occupational future time perspective, perceived remaining time

## Abstract

**Introduction:**

This study explores the relationships between occupational future time perspective (OFTP) dimensions—focus on opportunities, perceived remaining time, and focus on limitations—and employability among Chinese college students, with a particular focus on the mediating role of decent work perceptions.

**Method:**

The sample included 1,976 students from various universities across China.

**Results:**

The findings reveal that focus on opportunities and perceived remaining time are positively associated with employability, both directly and indirectly through higher perceptions of decent work. In contrast, focus on limitations shows a weak negative association with employability, both directly and via its association with lower perceptions of decent work.

**Discussion:**

These results indicate that future-oriented career thinking and perceptions of decent work are closely related to employability outcomes. Practical implications include the need for curriculum reforms that strengthen students’ proactive engagement in career planning and self-directed learning, while also promoting psychological resources that help them maintain positive outlooks. For college counselors, these findings underscore the value of fostering self-efficacy, promoting exploratory career behaviors, and helping students build realistic and optimistic perceptions of decent work to enhance their career readiness. By addressing these factors, Chinese colleges can better prepare students for meaningful and sustainable careers in a competitive labor market.

## Introduction

In an increasingly dynamic and competitive job market, the employability of college students has become a critical area of study, particularly for Chinese students navigating both academic and professional landscapes (Wen and Qing, 2024). Occupational future time perspective (OFTP) provides a valuable framework for understanding how perceptions of future career opportunities and time remaining in a career are related to employability (Zacher, 2013; Zacher and Frese, 2009). Additionally, the concept of decent work, which reflects fair, equitable, and fulfilling job conditions, is an essential construct in this relationship, offering insights into how students’ career outlooks interact with workplace expectations and are related to their readiness for the workforce (Ferraro et al., 2018a; Ferraro et al., 2018b). Beyond advancing theoretical understanding, this study has significant practical implications. Examining the relationship between OFTP, decent work perceptions and employability among Chinese college students contributes to addressing pressing concerns about youth unemployment and underemployment. In the context of China’s rapidly evolving labor market, insights from this research can inform career development programs and policies tailored to the needs of students transitioning into the workforce (Al-Fattal, 2025). In addition, rapid technological change and the growing impact of artificial intelligence have intensified the challenges students face when planning their careers (Wong, 2024).

Moreover, by highlighting the role of decent work perceptions, the study underscores the importance of creating equitable and fulfilling job opportunities that align with students’ aspirations (Wang et al., 2019). This alignment not only is expected to be associated with higher levels of individual employability but also with broader organizational outcomes, such as employee retention and productivity (Duffy et al., 2024). Educational institutions, policymakers, and employers can utilize these findings to design interventions that promote a more optimistic future time perspective while fostering an environment conducive to perceptions of decent work (Ma et al., 2021).

Recent literature suggests that perceptions of decent work may serve as a key psychological mechanism linking individuals’ views of their occupational future with their motivation and preparedness to enter the labor market (Duffy et al., 2016; Ferraro et al., 2018a). Specifically, when students anticipate meaningful and fair employment conditions, they may feel more confident and proactive in developing the competencies associated with employability. Conversely, a lack of belief in the attainability of decent work might dampen the motivational value of a future-oriented career perspective. By conceptualizing decent work perceptions as a mediator, this study builds on the Psychology-of-Working Theory (Duffy et al., 2016), which highlights the role of contextual and individual factors in shaping work-related outcomes through perceptions of access to decent employment. This approach offers a more nuanced understanding of how OFTP is associated with employability through students’ expectations of their future work conditions.

Although OFTP was initially conceptualized in the context of older workers and retirement-related decisions (Fasbender et al., 2019), the three dimensions of the construct remain theoretically relevant across the entire career span. For college students, a focus on opportunities reflects attention to exploration, growth, and the identification of emerging career possibilities. Perceived remaining time refers to students’ subjective sense of how much occupational time they believe they have ahead to invest in education, skill development, and career experimentation. Finally, focus on limitations captures concerns about constraints, narrowing options, or anticipated barriers that may restrict future career pathways. Applying OFTP to early career stage students therefore extends the construct to a younger developmental context and complements previous research conducted primarily with older or already employed populations.

Chinese college students represent a particularly relevant population for examining employability in the context of OFTP and perceptions of decent work. This group is navigating their transition to the workforce amid significant economic transformations, rising youth unemployment, and increasing competition in China’s labor market. Moreover, socioeconomic disparities—such as differences in Hukou status, family income, and parental education—shape students’ access to career resources and their perceptions of future employment prospects. Such structural inequalities are consistent with broader evidence showing that educational disparities and labor market outcomes often cluster across countries and social groups (Gaweł and Krstić, 2021). These contextual characteristics make Chinese college students not only a vulnerable group in terms of labor market integration but also a theoretically informative one for understanding how psychological constructs like OFTP and decent work perceptions operate across diverse backgrounds. Studying this group provides valuable insights into how future-oriented thinking and perceived work conditions are related to employability outcomes during a pivotal developmental stage.

Additionally, the study provides a nuanced understanding of how different dimensions of OFTP interact with perceptions of decent work. This perspective can guide targeted strategies to reduce the negative associations of focus on limitations with employability and enhance the positive associations of focus on opportunities and perceived remaining time with students’ employability-related outcomes. Ultimately, these insights have the potential to empower Chinese college students to achieve their career goals while navigating the complexities of the modern labor market.

## Literature review and hypothesis development

### OFTP and Chinese college students’ employability

Although OFTP was originally developed to examine age-related differences in occupational motivation among older workers, its dimensions can also be meaningfully applied to individuals at earlier career stages. For college students, OFTP reflects how they make sense of their emerging career opportunities, how much occupational time they perceive ahead of them, and the extent to which they anticipate barriers during their transition into the labor market. Extending OFTP to this younger population allows the construct to be examined from a life-span perspective, capturing how future-oriented thinking develops long before mid- or late-career stages. The dimension of focus on opportunities and a positive outlook on career growth and success is a key component of OFTP. Individuals with a strong focus on opportunities often engage in behaviors that enhance employability, such as building skills and developing professional networks. Research has demonstrated that such a perspective fosters proactive career development, making students better equipped for future employment opportunities (Zacher and Frese, 2009). For Chinese college students, this dimension is particularly important as they prepare to enter a competitive job market where optimism and strategic engagement with career development are critical.

The dimension of focus on opportunities within the OFTP is particularly significant because a future-oriented mindset fosters proactive behaviors that are crucial for navigating the job market. Ling et al. (2022) emphasize that students with a strong future orientation are more likely to engage in problem-based learning, which in turn promotes critical thinking, problem-solving, and skill acquisition—key components of employability in today’s evolving labor market. While the association between focus on opportunities and proactive career behaviors has been widely supported (Zacher and Frese, 2009), few studies have explicitly examined how this dimension of OFTP is related to employability outcomes through psychological mediators such as decent work perceptions. Moreover, much of the research has focused on employed adults, rather than students in transition to the labor market. Future research should further explore how opportunity-focused thinking interacts with students’ beliefs about their future work conditions.

Hence, the first hypothesis is:

*H1:* Focus on opportunities is directly and positively associated with Chinese college students’ future employability.

Another dimension of OFTP is perceived remaining time, which represents an individual’s subjective assessment of how much time they have left to achieve career goals. A longer perceived remaining time often encourages long-term planning and sustained investment in career development. Studies suggest that individuals who feel they have ample time to realize their goals are more likely to engage in future-oriented behaviors, which are associated with their employability (Zacher and Frese, 2009). In the context of Chinese college students, this perspective supports a focus on continuous learning and skill acquisition. The concept of perceived remaining time reflects how students assess their career timeline, which is related to their motivation and actions toward enhancing employability. Research emphasizes that a clear perception of remaining time fosters a positive future orientation, which is directly associated with higher perceived employability among students (Ling et al., 2022). This association is statistically mediated by problem-based learning, a process that enhances critical thinking and problem-solving skills (Janeiro et al., 2017). Students with a strong perception of remaining time are more likely to engage in activities such as skill development and career planning, aligning with labor market demands. Although perceived remaining time has been linked to motivation and long-term career planning, its role in being associated with employability among students remains underexplored. Existing research rarely addresses how this OFTP dimension interacts with contextual factors such as perceived labor market access or psychological constructs like decent work expectations. This study contributes to filling that gap by analyzing how time-related perceptions are related to students’ readiness for employment.

Furthermore, the second hypothesis is:

*H2:* Perceived remaining time is directly and positively associated with Chinese college students’ future employability.

On the other hand, focus on limitations, which involves preoccupation with barriers and constraints, can have a negative association with employability. This perspective often leads to reduced self-efficacy and disengagement from career-enhancing activities, undermining an individual’s ability to adapt and succeed in the job market. Evidence highlights that individuals with a focus on limitations are less likely to exhibit proactive career behaviors, which can be related to lower employability outcomes (Cheung et al., 2018). For Chinese college students, mitigating this negative outlook is crucial for promoting resilience and career readiness. The concept of OFTP highlights how individuals perceive their future in the context of their careers, encompassing dimensions such as focus on opportunities and limitations. A focus on limitations, as a negative dimension of future time perspective (FTP), has been shown to exacerbate career decision-making difficulties among Chinese college students (Ling et al., 2022). Students with a limited view of their future are more likely to struggle with informed career decisions, leading to adverse associations with their employability. Career decision-making difficulties arise because a negative future orientation often is related to lower motivation and self-belief, reducing students’ ability to navigate complex career pathways effectively.

Finally, the third hypothesis is:

*H3:* Focus on limitations is directly and negatively associated with Chinese college students’ future employability.

### The mediating role of decent work perceptions in the relationships between OFTP dimensions and students’ employability

Decent work perceptions—which reflect individuals’ evaluations of fair, secure, and fulfilling job conditions—serve as a psychological bridge between OFTP and employability. These perceptions are positively associated with a focus on opportunities and perceived remaining time and may be inversely related to a focus on limitations.

A focus on opportunities, characterized by future career clarity, proactive personality, and positive expectations, has been shown to be associated with greater belief in attaining decent and meaningful work. This belief in attaining perceptions of decent work, in turn, is associated with career exploration behaviors and the development of competencies that are central to employability. Students who maintain a strong focus on opportunities are more likely to perceive their future employment as equitable and fulfilling, which reinforces their engagement in employability-enhancing activities (Ling et al., 2022). Similarly, perceived remaining time - students’ belief that they still have a long and open future in their careers - can be related to enhanced motivational power of decent work perceptions. When students believe they have enough time to grow professionally, they are more likely to internalize decent work as an attainable and motivating standard for their future employment (Shiyuan et al., 2022). Conversely, a dominant focus on limitations—emphasizing obstacles and perceived time constraints—may be associated with weaker belief in the possibility of decent work and with reduced motivation to invest in career development and employability (Yan et al., 2023).

Recent empirical evidence supports the mediating role of decent work perceptions in various occupational contexts. For example, Fang et al. (2025) found that perceptions of decent work partially mediated the relationship between role clarity and job embeddedness in Chinese nurses. Wang et al. (2024) demonstrated that perceptions of decent work mediated the association between innovative behavior and structural empowerment. From an educational perspective, Wang et al. (2024) identified perceptions of decent work as a significant mediator between perceived social support and future career expectations among Chinese students. In a related context, Wen et al. (2023) examined the relationship between career calling and resilience in pre-service teachers and found that perceptions of decent work contributed to positive developmental outcomes. Finally, Zhang et al. (2024) found that decent work perceptions mediated the effect of effort–reward imbalance on well-being among psychiatric nurses. Although these studies do not focus directly on OFTP, they provide converging evidence that perceptions of decent work are shaped by individual traits and contextual variables, and in turn are associated with critical work and career outcomes. Building on the Psychology-of-Working Theory (Duffy et al., 2016), the present study proposes that decent work perceptions statistically mediate the associations between the three dimensions of OFTP and employability.

Despite growing attention to the concept of decent work, the existing literature has notable limitations. Most empirical studies on decent work perceptions have been conducted with working populations, such as nurses or employed graduates, leaving a gap in our understanding of how these perceptions develop among students preparing to enter the labor market (Fang et al., 2025; Wang et al., 2024). Moreover, while previous research has established links between future orientation and employability (Zacher, 2013), few studies have examined the role of decent work perceptions as a psychological mechanism that may help to account for this relationship. This omission is particularly relevant in the context of emerging economies, where students’ optimism about decent work opportunities may be shaped by structural labor market conditions. Additionally, little is known about how different OFTP dimensions relate differently to decent work perceptions, especially in student populations. These gaps highlight the need to investigate not only direct links between OFTP and employability, but also the nuanced, indirect pathways through which students’ expectations of fair and fulfilling work may be related to their career development. Addressing this gap can offer valuable insights for designing interventions that align students’ career expectations with the realities of the labor market.

*Specifically, it is hypothesized that decent work perceptions statistically mediate the associations between focus on opportunities and employability (H4), perceived remaining time and employability (H5), and focus on limitations and employability (H6).*

**Figure 1:**
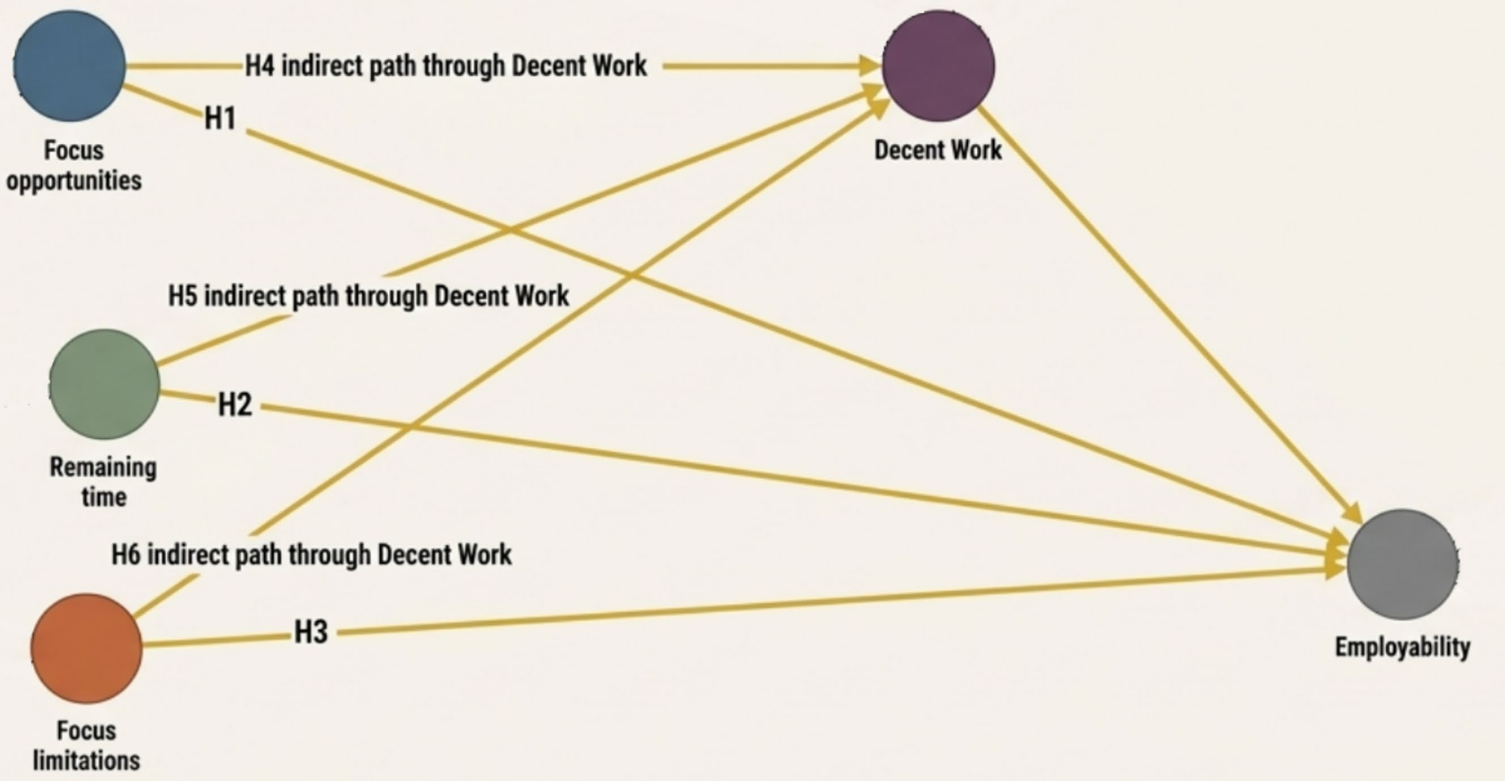
Conceptual model and hypotheses.

By exploring these dimensions of OFTP alongside the mediating influence of decent work perceptions, this study aims to uncover the mechanisms that drive employability among Chinese college students. This approach offers insights into targeted strategies that can enhance career development and ensure that students are well-prepared for future challenges in the labor market. The full set of hypotheses is displayed in Figure 1.

## Method

### Participants

The study included 1,976 Chinese college students representing a diverse range of demographic and socioeconomic backgrounds. Participants were aged between 18 and 25 years (*M* = 19.95, SD = 1.77). In terms of gender, 856 students identified as female (43.3%), and 1,120 students identified as male (56.7%).

Hukou status and family income were collected to allow for preliminary group comparisons and to examine whether the core study variables remained stable across major sociodemographic categories. Participants’ Hukou (household registration) status was diverse, with the majority holding an urban Hukou (74.0%, *n* = 1,462). Students with a rural Hukou accounted for 16.1% (*n* = 318) of the sample, while 3.0% (*n* = 60) belonged to the floating population, representing rural Hukou holders living in urban areas. Additionally, 6.5% (*n* = 129) had a converted Hukou status, indicating they had transitioned from rural to urban Hukou, and a small percentage (0.4%, *n* = 7) reported having another temporary residency status or no formal Hukou. Parental education levels were distributed across three categories. Most students reported coming from families where parents had moderate educational attainment (55.1%, *n* = 1,089), including secondary school or vocational education. Approximately 23.5% (*n* = 465) of students’ parents had low education levels, such as primary school or below. Finally, 21.4% (*n* = 422) of students reported having parents with high educational attainment, such as a college degree or above.

Family income was categorized into three levels. Nearly half of the participants (48.6%, *n* = 961) came from middle-income families, indicating stable financial situations with access to moderate resources. A significant portion (39.6%, *n* = 782) belonged to low-income families, often associated with rural or less developed regions. A smaller group (11.8%, *n* = 233) reported coming from high-income families, reflecting greater access to financial resources and opportunities. Participants were enrolled in a wide range of academic disciplines, with the largest groups being from Engineering and Technology (24.2%, *n* = 479), Economics and Business (21.8%, *n* = 431), and Medicine and Health Sciences (17.3%, *n* = 342). Smaller proportions were studying Education (15.7%, *n* = 310), Humanities and Social Sciences (6.6%, *n* = 131), Art and Design (6.3%, *n* = 125), Agriculture and Environmental Sciences (4.4%, *n* = 86), and Science (3.6%, *n* = 72). While the sample reflects a broad range of socioeconomic, demographic, and academic characteristics, it was obtained through convenience sampling and may not be representative of the general population of Chinese college students. Therefore, the findings should be interpreted with caution regarding their generalizability.

### Procedure

This study was approved by the Institutional Review Board of Dezhou University (Approval No. ID-55932), with formal approval granted on March 22nd, 2024. The approval process ensured that the study adhered to the ethical principles outlined in the Declaration of Helsinki and its subsequent updates. Participants were provided with detailed information about the study’s purpose, procedures, potential risks, and benefits before their involvement. Informed consent for participation and the publication of aggregated, anonymized data was obtained electronically. Participants were informed of their right to withdraw from the study at any time without any consequences and were assured that all responses would remain anonymous. The study employed a cross-sectional survey design to explore the relationships between OFTP, perceptions of decent work, and employability among Chinese college students. Data collection occurred over 1 month, starting in late January 2024, to account for the academic calendar and ensure accessibility before the Spring Festival holiday. Recruitment utilized convenience sampling, with survey dissemination achieved through partnerships with university groups, student organizations, and posts on widely used social media platforms such as WeChat and Weibo.

The survey was administered using Questionnaire Star, a secure and widely used commercial survey tool that ensured a user-friendly interface for participants and compliance with data protection standards. The survey link included a brief description of the study, eligibility criteria (Chinese college students aged 18–25), and an estimated completion time of 10–15 min. To ensure participant privacy and data protection, no personally identifiable information was collected. All responses were securely stored within the survey platform, which complies with international data protection standards. Only authorized members of the research team had access to the data, and all analyses were conducted using anonymized datasets. Findings are presented in aggregated form to uphold confidentiality and ethical research practices. This approach enabled the researchers to engage a diverse group of participants across various disciplines and geographic regions, providing rich data for the study’s objectives.

### Measures

*Occupational Future Time Perspective* was assessed using a Chinese version of the scale adapted by Lam et al. (2019) for the employment context, which is based on the work of Ho and Yeung (2016). This scale evaluates three distinct dimensions: perceived remaining time, focus on opportunities, and focus on limitations. These dimensions reflect different facets of individuals’ perspectives on their occupational future. Examples of the items include “My occupational future is filled with possibilities” (focus on opportunities) and “There is plenty of time left in my occupational life to make new plans” (perceived remaining time). For the Focus on limitations, items capture a more constrained perspective, such as “There are few opportunities in my occupational future.” Participants rated their agreement with each item on a 5-point Likert scale ranging from 1 (strongly disagree) to 5 (strongly agree). In line with prior research by Zacher (2013), item 5 (“I only have limited possibilities in my occupational future”) was excluded from this study due to consistently low factor loadings (−0.345) across the dimensions in exploratory factor analyses during the adaptation process. Nine items were used, and scores were calculated separately for each of the three subdimensions. Higher scores on focus on opportunities and perceived remaining time indicate a more expansive and optimistic occupational perspective. In comparison, higher scores on focus on limitations reflect a more constrained and restrictive outlook.

*Perceptions of Decent Work* were assessed using an adapted version of the Decent Work Perceptions Scale (DWPS), developed by Yan et al. (2023) to measure decent work among knowledge workers. The original 13-item scale evaluates four dimensions of decent work: work security, respect and support, self-value, and professional skills. This scale has demonstrated reasonable reliability and validity in previous research. For this study, nine items were selected based on their factor loadings in the confirmatory factor analysis from the original research, ensuring the strongest indicators of the construct were retained. To align with the study’s focus on occupational future time perspective, the items were adapted to assess participants’ perceptions of their *future* work environment. For example, items were rephrased as “My future job can provide security for my work” and “I can get help and support in my future work.” Participants responded to the items on a 5-point Likert scale ranging from 1 (strongly disagree) to 5 (strongly agree). Higher scores reflected stronger perceptions of decent future work across the four dimensions. This adaptation allowed for an assessment of participants’ forward-looking perceptions of decent work, consistent with the study’s objectives.

*Employability* was assessed using items from the scale developed by Rothwell et al. (2008). The scale focuses on students’ perceptions of their employability based on their educational background and external labor market conditions. The scale has demonstrated adequate psychometric properties with Western and Eastern samples (Rothwell et al., 2009). For this study, six items were selected from the original scale based on their factor loadings, with only those exceeding 0.50 included, as identified in the original study. The selected items reflect key dimensions of employability, such as the reputation of the university, the labor market demand for graduates from the students’ field, and the competitiveness of their degree programs. Sample items include “Employers are eager to employ graduates from my university,” “The status of this university is a significant asset to me in job seeking,” and “People in the career I am aiming for are in high demand in the external labor market.” Additionally, the item “I feel I could get any job so long as my skills and experience are reasonably relevant” was included to assess self-perceived employability. Participants rated their agreement with each item on a 5-point Likert scale ranging from 1 (strongly disagree) to 5 (strongly agree). Higher scores indicated a greater perception of employability based on their educational and professional context. The measure has demonstrated sufficient reliability in studies with Chinese university student samples (Cheung et al., 2018; Yizhong et al., 2017), supporting its use in the current research. This approach ensured that the scale remained reliable and aligned with its validated factor structure. All the factor loadings of the items are included in Supplementary Table S1.

### Data analytic strategy

Data analysis was conducted using a combination of statistical software to address the research objectives. SPSS (version 29.0) was used to calculate descriptive statistics and perform Pearson’s correlation analyses. Descriptive statistics, including means and standard deviations, provided an overview of the distribution and central tendencies of the key variables. At the same time, correlation analyses assessed the strength and direction of relationships among the variables. To test the hypothesized relationships, including direct, indirect, and mediated effects, the study employed the SmartPLS 4 (Ringle et al., 2024). This software is particularly well-suited for exploratory and predictive modeling, especially when analyzing complex models with latent constructs. The Partial Least Squares (PLS) algorithm was used to estimate path coefficients and evaluate the significance of the relationships between variables. A bootstrapping procedure with 5,000 resamples was applied to assess the significance of direct and indirect effects, providing robust non-parametric confidence intervals. The 95% bias-corrected confidence intervals were used to determine significance, with effects considered significant if the confidence interval did not include zero. The combined use of SPSS for descriptive and correlational analyses and SmartPLS for SEM allowed for a comprehensive examination of the relationships among OFTP, decent work perceptions, and employability. These methods provided detailed insights into both the direct and indirect pathways within the conceptual model. Before estimating the structural model, preliminary analyses were conducted to examine whether the main constructs differed across key sociodemographic groups. Specifically, one-way ANOVAs were performed to test group differences in employability, perceptions of decent work, and the three OFTP dimensions as a function of hukou status and family income. These analyses allowed the assessment of the stability of the core variables across background characteristics prior to estimating the SEM.

## Results

### Descriptive statistics and correlations

Descriptive statistics for the key study variables were calculated, including the dimensions of OFTP (focus on opportunities, perceived remaining time, and focus on limitations), perceptions of decent work, and employability. The mean scores indicate that focus on opportunities (*M* = 3.48) and perceptions of decent work (*M* = 3.57) were above the scale midpoint (3.00), suggesting generally favorable evaluations in these areas. In contrast, employability (*M* = 3.42) was only slightly above the midpoint, while focus on limitations (*M* = 2.57) and perceived remaining time (*M* = 2.31) fell below it. These results suggest that, on average, students perceived fewer remaining years in their occupational future and reported a moderate level of perceived constraints.

Standard deviations indicate moderate variability across all variables, with perceived remaining time (SD = 1.23) and focus on limitations (SD = 1.12) showing the highest dispersion, reflecting a broader diversity of responses on these dimensions. Table 1 provides detailed descriptive statistics and correlations for all variables.

**Table 1:** Descriptive statistics and Pearson’s correlations (*N* = 1976).

Variable	*M*	SD	1	2	3	4	5
1. Focus on Opportunities	3.48	0.97	--				
2. Remaining Time	2.31	1.23	0.275**	--			
3. Focus on Limitations	2.57	1.12	−0.495**	−0.095**	--		
4. Perceptions of Decent Work	3.57	0.74	0.515**	0.232**	−0.261**	--	
5. Employability	3.42	0.91	0.423**	0.350**	−0.250**	0.690**	--

**p < 0.001.

In sum, the descriptive statistics and correlation patterns suggest that focus on opportunities and perceptions of decent work are positively associated with employability, while focus on limitations shows a weaker or negative relationship. These preliminary associations provide initial support for the hypothesized model and justify further examination through the measurement and structural model assessments presented in the following sections.

### Employability and decent work differences across background variables

Although no group differences were hypothesized, preliminary analyses were conducted to examine whether the main study variables varied according to key sociodemographic characteristics. A t-test revealed no significant difference in employability between male (*M* = 3.41, SD = 0.95) and female students (*M* = 3.43, SD = 0.88), *t*(1974) = −0.291, *p* = 0.386. One-way ANOVAs also showed no significant differences in employability based on family income, *F*(2, 1973) = 1.873, *p* = 0.154, or parental education, *F*(2, 1973) = 0.690, *p* = 0.500. Taken together, these results suggest that employability perceptions were relatively stable across these sociodemographic subgroups.

To further assess the stability of the core constructs across contextual factors that are especially relevant in the Chinese labor-market context, additional one-way ANOVAs were conducted by hukou status (five categories) and family income (three levels). Employability did not differ significantly across hukou groups, *F*(4, 1971) = 2.241, *p* = 0.062, and perceptions of decent work showed the same pattern of non-significant differences, *F*(4, 1971) = 1.794, *p* = 0.127. Similarly, perceptions of decent work did not differ significantly across income groups, *F*(2, 1973) = 2.585, *p* = 0.076. Focus on limitations also showed no significant differences across hukou, *F*(4, 1971) = 0.746, *p* = 0.560, or income, *F*(2, 1973) = 2.998, *p* = 0.050.

In contrast, small but statistically significant differences emerged for some OFTP dimensions. Focus on opportunities differed across hukou status, *F*(4, 1971) = 3.221, *p* = 0.012, and perceived remaining time showed statistically significant differences for both hukou, *F*(4, 1971) = 4.658, *p* < 0.001, and income, *F*(2, 1973) = 3.204, *p* = 0.041. However, the associated effect sizes were very small (η^2^ ≤ 0.009), indicating that these group differences are modest in practical terms. Overall, these preliminary analyses indicate that employability and perceptions of decent work remain relatively stable across background variables, while some OFTP dimensions vary slightly by hukou status and family income.

### Measurement model

To ensure the validity and reliability of the constructs used in the study, a measurement model was evaluated prior to testing the hypotheses. The measurement model assesses the psychometric properties of the latent variables by examining construct reliability, convergent validity, and discriminant validity. Construct reliability was evaluated using Cronbach’s alpha and composite reliability (rho_a and rho_c), ensuring that each construct demonstrated consistent internal measurement. Convergent validity was established through the average variance extracted (AVE), which measures the amount of variance captured by the construct in relation to the variance attributable to measurement error. Discriminant validity was examined using the Heterotrait-Monotrait (HTMT) ratio and the Fornell-Larcker criterion, both of which confirm that the constructs are sufficiently distinct from one another. Lastly, collinearity statistics were analyzed to confirm the absence of multicollinearity issues among the indicators, ensuring the robustness of the subsequent structural model analysis. This rigorous evaluation supports the reliability and validity of the measurement instruments used in the study.

### Construct reliability and validity

The reliability and validity of the constructs were assessed using Cronbach’s alpha, composite reliability (rho_a and rho_c), and average variance extracted (AVE), as Table 2 shows. All constructs demonstrated acceptable internal consistency, with Cronbach’s alpha and composite reliability exceeding the threshold of 0.7. Furthermore, AVE values for each construct were above the recommended threshold of 0.5, indicating adequate convergent validity.

**Table 2:** Reliability and convergent validity.

Construct	Cronbach’s alpha	Composite reliability (rho_a)	Composite reliability (rho_c)	Average variance extracted (AVE)
Perceptions of decent work	0.883	0.892	0.905	0.515
Employability	0.910	0.912	0.930	0.690
Focus on limitations	0.790	0.791	0.905	0.826
Focus on opportunities	0.895	0.902	0.928	0.762
Perceived remaining time	0.915	0.917	0.947	0.856

### Discriminant validity

Discriminant validity was assessed using the Heterotrait-Monotrait (HTMT) ratio and the Fornell-Larcker criterion, as Tables 3, 4 show. The HTMT values for all construct pairs were below the threshold of 0.85, providing evidence of discriminant validity. Similarly, the Fornell-Larcker criterion was satisfied, as each construct’s square root of the AVE exceeded its correlations with other constructs.

**Table 3:** Discriminant validity (HTMT ratio).

Construct pair	HTMT value
Employability ↔ Perceptions of decent work	0.766
Focus on limitations ↔ Perceptions of decent work	0.312
Focus on limitations ↔ Employability	0.296
Focus on opportunities ↔ Perceptions of decent work	0.575
Focus on opportunities ↔ Employability	0.466
Focus on opportunities ↔ Focus on limitations	0.591
Perceived remaining time ↔ Perceptions of decent work	0.260
Perceived remaining time ↔ Employability	0.376
Perceived remaining time ↔ Focus on limitations	0.111
Perceived remaining time ↔ Focus on opportunities	0.303

**Table 4:** Fornell-Larcker criterion.

Construct	Decent work	Employability	Focus on limitations	Focus on opportunities	Remaining time
Perceptions of decent work	0.718				
Employability	0.703	0.831			
Focus on limitations	−0.277	−0.252	0.909		
Focus on opportunities	0.530	0.423	−0.494	0.873	
Perceived remaining time	0.243	0.342	−0.095	0.275	0.925

### Multicollinearity

To assess multicollinearity among the indicators, Variance Inflation Factor (VIF) values were examined. All indicators showed VIF values below 6.1, with most values falling under 3.7. Although earlier guidelines proposed a critical threshold of 10, more conservative criteria are now commonly adopted in psychological and educational research, where VIF values below 5—or even below 3—are recommended to ensure robustness (Hair et al., 2019; Kock and Lynn, 2012). In this study, only two indicators marginally exceeded the threshold of 5 (RT1 = 5.839; RT2 = 6.032), while the rest remained well below. Given the theoretical relevance of the items and the absence of extreme collinearity, no indicators were removed. A full list of factor loadings is provided in Supplementary Table S1, and the VIF values are included as Supplementary Table S2.

### Structural model

#### Model fit and model quality

The structural model was evaluated using multiple fit indices to determine its overall quality and predictive relevance. The Standardized Root Mean Square Residual (SRMR) for both the saturated model and the estimated model was 0.066, indicating a good fit, as values below 0.08 are considered acceptable. Additional metrics, including d_ULS (1.301) and d_G (0.477), further support the adequacy of the model. The Chi-square statistic (5632.695) and the Normed Fit Index (NFI, 0.823) were also within acceptable ranges, suggesting that the hypothesized structural relationships align well with the observed data. The model’s predictive power was assessed using the R-squared values of the dependent constructs. Perceptions of Decent Work explained 29.1% of the variance (adjusted *R*-squared = 0.290), while Employability accounted for 52.8% of the variance (adjusted *R*-squared = 0.528). These results indicate moderate to substantial predictive accuracy for the endogenous variables, demonstrating that the model effectively explains a significant proportion of variance in both constructs. The f-squared effect size was used to evaluate the relative contribution of individual constructs to explaining variance in the dependent variables. Perceptions of Decent Work had a substantial effect on Employability (*f*^2^ = 0.616), highlighting its central statistical association with employability outcomes. Focus on Opportunities exhibited a moderate effect on Perceptions of Decent Work (*f*^2^ = 0.236), indicating that an opportunity-oriented occupational perspective is more strongly associated with higher perceptions of decent work. Perceived Remaining Time had a moderate effect on Employability (*f*^2^ = 0.062), further underscoring association with employability perceptions. In contrast, Focus on Limitations demonstrated negligible effects on Perceptions of Decent Work and Employability, with *f*^2^ values of 0.001 and 0.005, respectively. These findings emphasize the strong associations of Perceptions of Decent Work and Focus on Opportunities in the structural model, while the role of Focus on Limitations appears minimal.

Predictive relevance was assessed using PLSpredict with Q^2^predict values for the observed indicators. The results demonstrated that most indicators exhibited positive Q^2^predict values, confirming the model’s predictive accuracy. Among the Perceptions of Decent Work indicators, DW6 (Q^2^predict = 0.282) and DW9 (Q^2^predict = 0.240) displayed the highest predictive relevance. For Employability, Employability2 (Q^2^predict = 0.208) and Employability1 (Q^2^predict = 0.175) showed the strongest predictive performance. These values suggest that the model effectively predicts both constructs, with certain indicators contributing more substantially to the predictive power. In summary, the structural model demonstrated a strong overall fit, moderate to substantial predictive power, and reliable predictive accuracy for individual indicators, reinforcing the robustness of the hypothesized relationships. The results underscore the centrality of Perceptions of Decent Work and Focus on Opportunities in understanding employability outcomes and the model’s predictive capability in this context.

### Model selection criteria

The Bayesian Information Criterion (BIC) was calculated to compare the fit of the model’s constructs. Employability had a BIC value of −1450.754, while Perceptions of Decent Work had a slightly higher BIC value of −652.395. Since lower BIC values indicate better model fit, these results suggest that the model accounts for variance in employability more effectively compared to Decent Work.

The hypotheses were evaluated based on path coefficients, indirect effects, and total effects, with specific attention given to the relationships outlined in the theoretical model. The results are detailed below.

### Hypotheses testing

The direct effects provide insight into the immediate relationships between constructs. The path from Perceptions of Decent Work to Employability was significant and positive, indicating a strong direct relationship between these variables. Focus on Opportunities had a substantial positive effect on Perceptions of Decent Work but showed only a negligible direct effect on Employability. Similarly, Perceived Remaining Time was positively associated with Perceptions of Decent Work and Employability, though the effect on Employability was more pronounced. Focus on Limitations showed a weak and negative direct effect on both Perceptions of Decent Work and Employability. Although statistically significant, the magnitude of this relationship was small, indicating a limited role in a limited role in being associated with students’ perceptions of decent work and their sense of employability. These results suggest that a stronger focus on constraints may be associated with slightly lower expectations of future work quality and career readiness, though the practical impact appears minimal.

#### Indirect effects

The mediation effects were examined to test whether Perceptions of Decent Work mediated the relationships between the OFTP dimensions (Focus on Opportunities, Perceived Remaining Time, and Focus on Limitations) and Employability. The specific indirect effects revealed that Perceptions of Decent Work significantly mediated the positive relationship between Focus on Opportunities and Employability, as evidenced by a notable indirect effect. Perceived Remaining Time also exhibited a positive indirect effect on Employability through Perceptions of Decent Work. Focus on Limitations also demonstrated a small negative indirect effect on Employability through Perceptions of Decent Work. While the mediation path was statistically significant, the effect size was modest, suggesting that the indirect statistical association of Focus on Limitations operates with limited strength in the context of this model.

#### Total effects

The total effects combine both direct and indirect effects to provide a comprehensive view of the relationships. Focus on Opportunities emerged as the strongest positive correlate of Perceptions of Decent Work and Employability, with a significant total effect. Perceived Remaining Time also showed a moderate total effect on Employability, highlighting its importance in being associated with employability perceptions through its direct and indirect pathways. The total effect of Focus on Limitations on Employability remained weak and negative. These findings, as Table 5 shows, indicate that, compared to the other OFTP dimensions, Focus on Limitations plays a relatively minor role in being associated with employability outcomes, either directly or through perceptions of decent work.

**Table 5:** Summary of direct, indirect, and total effects with 95% bias-corrected confidence intervals.

Direct effects	*β*	95% BC CI
Perceptions of decent work → Employability	0.640	[0.604, 0.672]
Focus on limitations → Perceptions of decent work	−0.026	[−0.073, 0.021]
Focus on limitations → Employability	−0.054	[−0.093, −0.017]
Focus on opportunities → Perceptions of decent work	0.488	[0.440, 0.533]
Focus on opportunities → Employability	0.008	[−0.036, 0.055]
Perceived remaining time → Perceptions of decent work	0.106	[0.063, 0.149]
Perceived remaining time → Employability	0.180	[0.146, 0.213]

Specific indirect effects (via Perceptions of Decent Work)	*β*	95% BC CI
Focus on limitations → Perceptions of decent work → Employability	−0.017	[−0.047, 0.013]
Focus on opportunities → Perceptions of decent work → Employability	0.312	[0.276, 0.346]
Perceived remaining time → Perceptions of decent work → Employability	0.068	[0.041, 0.095]

Total effects	*β*	95% BC CI
Perceptions of decent work → Employability	0.640	[0.604, 0.672]
Focus on limitations → Perceptions of decent work	−0.026	[−0.073, 0.021]
Focus on limitations → Employability	−0.071	[−0.123, −0.021]
Focus on opportunities → Perceptions of decent work	0.488	[0.440, 0.533]
Focus on opportunities → Employability	0.320	[0.267, 0.370]
Perceived remaining time → Perceptions of decent work	0.106	[0.063, 0.149]
Perceived remaining time → Employability	0.248	[0.205, 0.290]

β represents standardized path coefficients. Confidence intervals correspond to 95% bias-corrected (BC) intervals obtained through bootstrapping. Indirect effects refer to mediation through Perceptions of Decent Work. Total effects represent the sum of direct and indirect effects.

**Figure 2:**
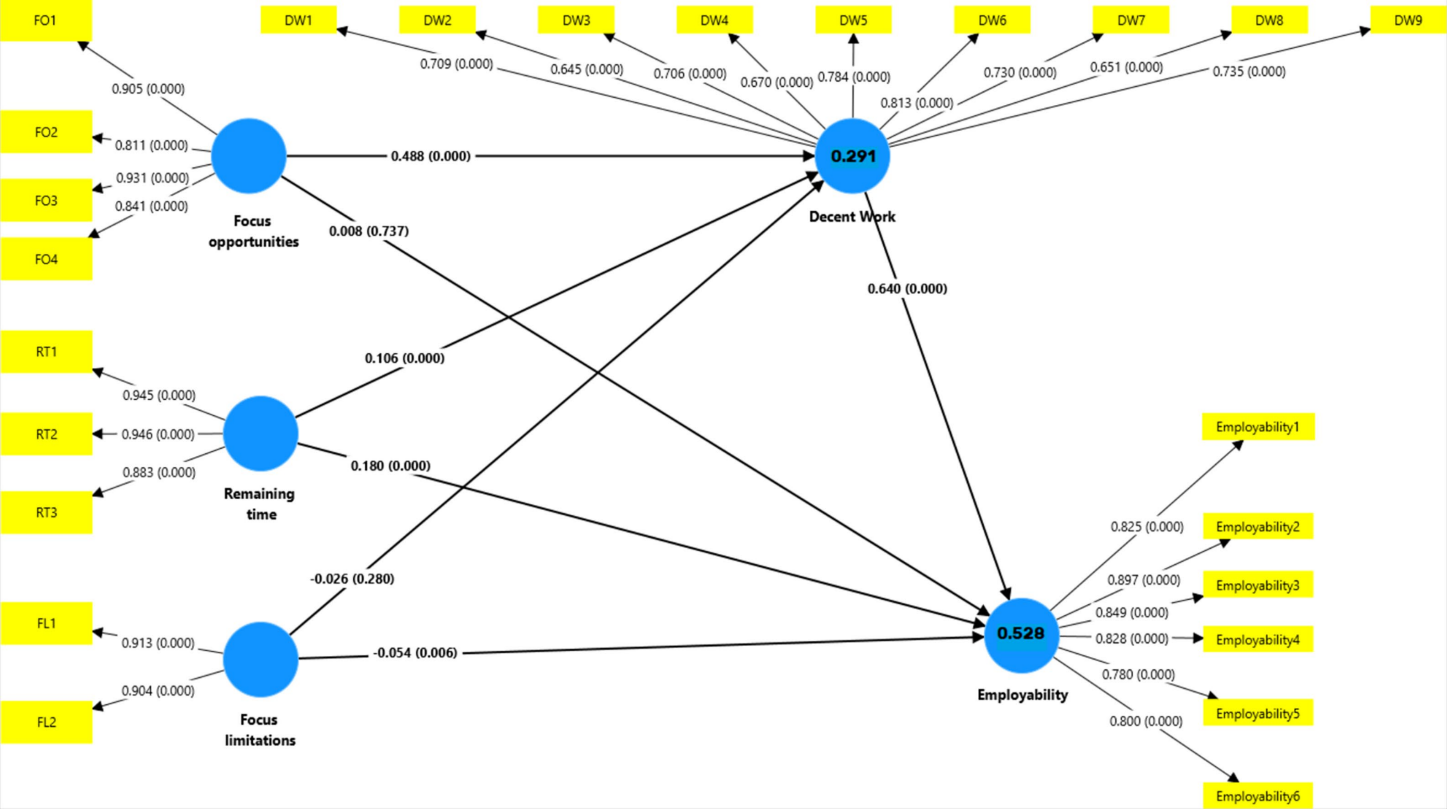
Path coefficients for all the variables in the model and factor loadings of the measurement model.

Figure 2 displays the path coefficients for all the variables in the Model and the factor loadings of the measurement model.

The path analysis showed that Perceptions of Decent Work had a significant and positive association with Employability (*β* = 0.640, *p <* 0.001). Focus on Opportunities was significantly associated with Perceptions of Decent Work (*β* = 0.488, *p <* 0.001), although its direct association with Employability was not significant (*β* = 0.008, *p =* 0.789). Perceived Remaining Time showed significant positive direct associations with Perceptions of Decent Work (*β* = 0.137, *p <* 0.001) and Employability (*β* = 0.092, *p <* 0.001). In contrast, Focus on Limitations showed no significant direct associations with Perceptions of Decent Work (*β* = −0.024, *p =* 0.254) or Employability (*β* = −0.030, *p =* 0.125).

Regarding indirect effects, Perceptions of Decent Work significantly mediated the associations between Focus on Opportunities and Employability (*β* = 0.312, *p <* 0.001) and between Perceived Remaining Time and Employability (*β* = 0.068, *p < 0*.001). The indirect association between Focus on Limitations and Employability through Perceptions of Decent Work was statistically significant but very small (*β* = −0.006, *p =* 0.032).

Taken together, these results support the indirect pathways specified in H4 and H5 and provide partial support for H6, while the direct associations specified in H1–H3 receive mixed support.

### Importance-performance map analysis

The Importance-Performance Map Analysis (IPMA) was conducted to assess the relative importance and performance of the constructs in being associated with Employability. This approach helps identify constructs that are both critical for outcomes (importance) and areas for improvement (performance), offering practical insights for intervention and strategy.

**Figure 3:**
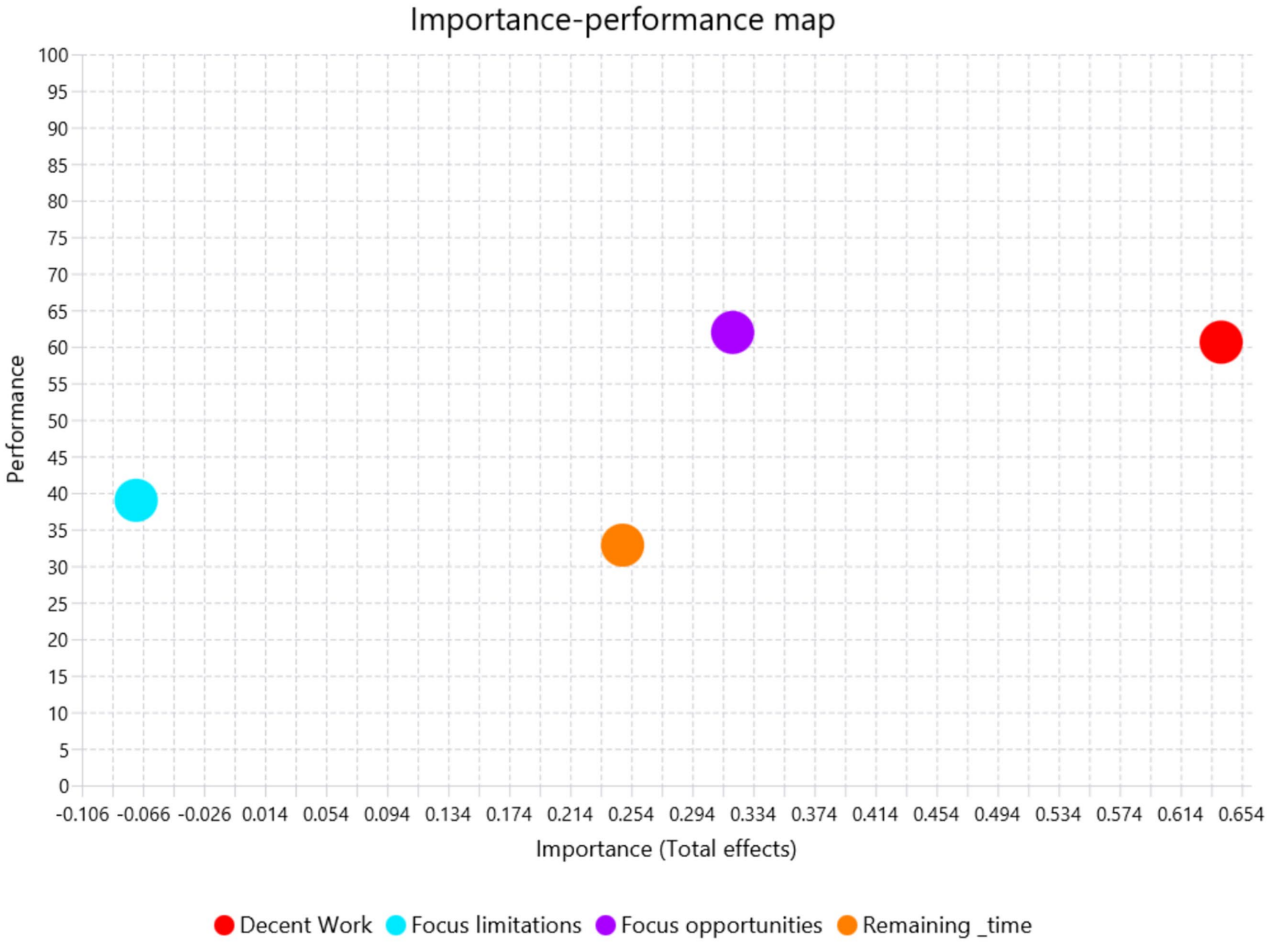
Importance-performance map predicting employability outcome.

The total effects of the constructs on Employability indicate their relative importance. Perceptions of Decent Work had the highest importance (total effect = 0.640), signifying its critical statistical association with Employability outcomes. Focus on Opportunities followed with moderately strong importance (total effect = 0.320), highlighting its substantial association with employability perceptions, as Figure 3 shows. Perceived Remaining Time showed moderate importance (total effect = 0.248), indicating that perceptions of remaining career time also play a role in in being associated with Employability. In contrast, Focus on Limitations exhibited negligible and negative importance (total effect = −0.071), suggesting that it detracts slightly from Employability but is not a major statistical driver in the model.

The performance scores reveal how well each construct performs in the context of Employability. Focus on Opportunities demonstrated the highest performance (61.959), closely followed by Perceptions of Decent Work (60.623), indicating that these constructs are not only important but also performing well in their associations with Employability. Perceived Remaining Time exhibited a lower performance score (32.857), suggesting room for improvement in leveraging this construct to enhance Employability outcomes. Focus on Limitations showed the lowest performance (38.990), reinforcing its minimal role in in being statistically related to Employability and highlighting its limited practical utility in this context.

The IPMA results suggest that strategies aiming to enhance Employability should prioritize maintaining and improving both the importance and performance of Perceptions of Decent Work and Focus on Opportunities. These two constructs not only have the strongest associations with Employability but are also performing well, making them critical leverage points for interventions. Additionally, while Perceived Remaining Time has moderate importance, its lower performance score indicates that targeted interventions could further enhance its role in being associated with Employability. In contrast, Focus on Limitations has limited importance and performance, suggesting it is not a priority area for improvement. Overall, the IPMA highlights the centrality of Perceptions of Decent Work and Focus on Opportunities in understanding Employability and underscores the potential to improve Perceived Remaining Time’s contribution to employability outcomes. These insights offer actionable guidance for enhancing students’ employability by focusing on key OFTP dimensions.

## Discussion

This study aimed to test the direct associations of OFTP dimensions on employability among Chinese College students and the mediating role of Perceptions of Decent Work in the relationship. Most of the proposed hypotheses have been supported, but some results deserve additional discussion.

### Focus on opportunities and employability

The hypothesis posited that focusing on opportunities would be directly and positively associated with Chinese college students’ future employability. The results, however, indicated that while focus on opportunities had a substantial positive effect on perceptions of decent work, its direct effect on employability was negligible. This finding suggests that the relationship between focus on opportunities and employability is primarily reflected in indirect pathways mediated by perceptions of decent work and other factors.

A future-oriented perspective has been consistently associated with proactive attitudes and goal-directed behaviors, which are relevant for career development (Zacher, 2013). In this context, fostering a focus on opportunities may be associated with helping students clarify their professional goals and engage more actively in behaviors that enhance employability via improved perceptions of future work quality. In addition, future career clarity has been found to help students leverage personal and social resources more effectively (Wang et al., 2022), reinforcing the relevance of this dimension of OFTP in preparing for labor market entry.

It is also important to consider that contextual factors may be associated with how future orientation relates to employability. For instance, the COVID-19 pandemic and related economic uncertainty may have disrupted students’ perceptions of opportunity, potentially moderating the strength of the association between this OFTP dimension and employability (Tian, 2023; Van der Heijden et al., 2019). These external conditions could partly explain why the direct effect of focus on opportunities was not statistically strong in the current data. Future research should further examine how broader societal and economic factors interact with OFTP and are associated with employability outcomes.

In summary, focus on opportunities did not exert a direct effect on employability but was significantly related to Perceptions of Decent Work, which in turn was associated with employability. This underscores the value of promoting future-oriented thinking as part of a broader framework that includes perceived work quality and structural career resources.

### Perceived remaining time and employability

The hypothesis posited that perceived remaining time would be directly and positively associated with Chinese college students’ future employability. The results support this hypothesis, showing that perceived remaining time is positively associated with both perceptions of decent work and employability, with the association with employability being more pronounced. This highlights the significant role of the perceived remaining time as a dimension of OFTP in being associated with employability outcomes.

A future-oriented outlook grounded in the perception of ample time remaining in one’s career may be associated with enhanced students’ motivation and engagement in career-related activities such as networking, skill-building, and academic achievement (Steindórsdóttir et al., 2024). This sustained engagement is likely to support long-term career development and be related to improved employability prospects (Soares et al., 2024). However, individual differences and contextual factors—such as socioeconomic background and access to institutional resources—can shape how students perceive their future career timeline (Tian, 2023). For instance, students facing financial hardship or limited support networks may perceive their future time more narrowly, potentially limiting their capacity to invest in career development.

Although these contextual influences were not directly tested in the present model, they offer important avenues for future research. Understanding how structural constraints interact with OFTP can help identify barriers to employability and guide targeted interventions. Future studies could explore whether access to career resources or perceived economic security moderates the association between perceived remaining time and employability.

In summary, perceived remaining time shows a significant and direct positive association with employability and contributes to enhanced perceptions of decent work. These findings underscore the importance of fostering long-term future orientation as part of employability development initiatives for Chinese college students.

### Focus on limitations and employability

The hypothesis posited that a focus on limitations would be negatively associated with Chinese college students’ future employability. The results supported this expectation, although the effect was statistically significant yet weak. This suggests that students who emphasize perceived constraints and time scarcity may report slightly lower levels of employability and perceive fewer prospects for decent work. However, the modest strength of the association indicates that this dimension of OFTP plays a relatively minor role in being associated with employability outcomes, particularly when compared to the stronger positive associations of focus on opportunities and perceived remaining time.

The limited association of focus on limitations may reflect the presence of unmeasured psychological or contextual variables that buffer its statistical relationship. For example, students may acknowledge constraints in their future careers without necessarily internalizing them as barriers to employability. This could be due to personal dispositions or institutional factors not captured in the current model. Although the present study did not examine such variables empirically, prior literature suggests that constructs like perceived support or optimism might mitigate the strength of the association between negative future perspectives and employability (Yeung and Ho, 2020).

Future research should investigate whether specific protective factors moderate the association between focus on limitations and employability. Longitudinal or mixed-method designs could also help to clarify the conditions under which a strong focus on limitations becomes more consequential for students’ career development.

In conclusion, focus on limitations exhibits a weak negative association with employability, and its role within the model should be interpreted with caution. While conceptually relevant, its practical impact appears modest, and further research is needed to explore potential moderators and contextual influences that may shape its associations.

### Perceptions of decent work as a mediator

The mediation effects of perceptions of decent work were examined across the three dimensions of OFTP: focus on opportunities, perceived remaining time, and focus on limitations. The findings revealed distinct mediated pathways, with Perceptions of Decent Work significantly associated with higher employability for focus on opportunities and perceived remaining time, while showing a small, negative mediating effect for focus on limitations.

The relationship between focus on opportunities and employability was significantly mediated by perceptions of decent work, highlighting the pivotal role of these perceptions in translating future-oriented career mindsets into employability outcomes. For Chinese college students, this suggests that educational strategies that promote proactive engagement with career planning—such as goal-setting, skill development, and guided reflection—may be associated with enhanced perceptions of decent work and with support for their employability (Shen et al., 2024). Moreover, perceptions of decent work may reduce employment-related uncertainty, encouraging students to plan more confidently for their future careers (Dinis et al., 2024; Xu et al., 2022).

Similarly, the mediating role of perceptions of decent work was significant in the relationship between perceived remaining time and employability. Students who perceive ample time left in their careers tend to adopt a more optimistic view of their professional development, which may strengthen their belief in the attainability of equitable and fulfilling work. This positive alignment supports sustained engagement in long-term planning and goal-oriented behavior (Duffy et al., 2021).

In contrast, the mediation effect of perceptions of decent work for focus on limitations was small and negative, indicating that a constrained future outlook may be associated with hindered development of favorable perceptions of future employment (Wan and Duffy, 2023). Students who primarily focus on constraints may struggle to envision their future work as fair or meaningful, which could be related to weaker motivation to prepare actively for the labor market (Nam and Kim, 2019; Seol et al., 2024). Although the present study did not examine potential moderating or buffering variables, prior literature suggests that these dynamics could be explored further in future research.

The total effects revealed that focus on opportunities emerged as the strongest positive correlate of both perceptions of decent work and employability, combining substantial direct and indirect pathways (Yizhong et al., 2017). Perceived remaining time also showed a moderate total effect, underscoring its contribution to both direct and mediated employability outcomes (Potts, 2022). In contrast, focus on limitations exhibited a weak and negative total effect, reinforcing its modest but adverse role in the model.

Taken together, the mediation effects of perceptions of decent work reveal differentiated mechanisms through which OFTP dimensions are statistically associated with employability. While future-oriented perspectives are associated with strengthened expectations of decent work and, in turn, higher perceived employability, a stronger focus on constraints tends to be associated with undermined perceptions. Future studies might explore additional mechanisms or moderators that shape these pathways and identify strategies for reinforcing positive expectations about students’ future work environments.

### Limitations and suggestions for future research

This study has several limitations that should be acknowledged. First, the data were collected using a cross-sectional design, which limits the ability to draw causal inferences about the relationships between OFTP, perceptions of decent work, and employability. Future research could employ longitudinal designs to explore how these relationships evolve, providing a clearer understanding of the potential causal pathways and possible reciprocal effects among these variables (Seol et al., 2024; Smith et al., 2020). Second, the sample was drawn using a convenience sampling approach, relying on Chinese college students recruited through university groups and social media platforms. Although this strategy enabled access to a large and demographically diverse sample, it introduces potential self-selection and sampling biases. As such, the sample cannot be considered representative of the general population of Chinese college students, and the findings should be interpreted with caution regarding their generalizability. Future research should consider adopting probabilistic or stratified sampling methods to enhance the external validity of the results and ensure broader applicability across regions, institutions, and demographic subgroups.

Third, though validated in prior research and adapted appropriately, the measures used in this study relied on self-reported data, which may introduce common method bias or social desirability effects. Incorporating additional data sources, such as employer assessments or objective measures of employability outcomes, could provide a more comprehensive and robust understanding of the constructs (Ayvaz and Karacan-Özdemir, 2024; Chu et al., 2021).

Fourth, while the study adapted the Decent Work Perceptions Scale to assess future work perceptions, the use of a scale originally developed for knowledge workers might limit its applicability to broader occupational contexts. Future research could further validate the adapted scale in diverse employment contexts to ensure its reliability and validity across different samples and settings. Fifth, although the study collected detailed information on students’ socioeconomic background, academic discipline, and hukou status, these contextual variables were not included as controls in the structural equation model. As a result, the findings may be subject to confounding effects, since these factors are known to be associated with both perceptions of career opportunities and employability outcomes. Future research should consider including such contextual variables as covariates in the analytical models to better isolate the unique contributions of psychological constructs such as OFTP and perceptions of decent work.

Lastly, the study focused exclusively on Chinese college students, which provides valuable insights into this population but limits the applicability of the findings to other cultural and educational systems. Given the cultural specificity of OFTP and perceptions of decent work, future studies could explore these constructs in other cultural settings to examine the universality or cultural specificity of the findings. Addressing these limitations in future research would contribute to a more nuanced and comprehensive understanding of the relationships between OFTP, perceptions of decent work, and employability. This would also enhance the theoretical and practical relevance of the findings for diverse populations and contexts.

### Practical implications for Chinese college curricula design

The findings of this study have important implications for Chinese college curricula aimed at enhancing student employability. First, future-oriented career education should be integrated into academic programs, with an emphasis on helping students identify long-term goals and engage in structured career planning. Similar to the present findings, recent research emphasizes the value of structured training programs aimed at promoting sustainable employment for young people (de Sousa and Romana, 2024). Instructional approaches such as problem-based learning and critical reflection can support the development of proactive career behaviors that are associated with students’ focus on opportunities and perceived remaining time (Renz and Vogel, 2024).

Second, promoting positive perceptions of decent work is essential. Curriculum modules that address fair labor conditions, workplace rights, and meaningful employment expectations can help students set realistic goals and reduce employment-related anxiety. These strategies can encourage students to seek fulfilling work that matches their values and capabilities.

Finally, integrating psychological support into the curriculum—such as workshops focused on self-efficacy, stress management, and goal setting—can help students counteract the potential negative impact of a strong focus on limitations and build confidence in their career potential.

### Implications for Chinese college counselors

College counselors play a crucial role in translating these findings into actionable support for students. Counselors should promote future-oriented thinking by encouraging students to focus on opportunities and perceived remaining time, helping them set clear goals and break them down into achievable steps.

Individual counseling sessions can reinforce proactive behaviors such as networking, skill-building, and self-assessment. For students with a strong focus on limitations, counselors should offer targeted interventions that build self-efficacy—through role-playing, guided reflection, and recognition of past achievements.

Incorporating discussions on perceptions of decent work into career counseling can help students align their aspirations with realistic job market opportunities. Counselors should also provide resources—such as access to career fairs, mentorship, and job search tools—to address contextual barriers and expand students’ sense of agency. Collaboration between counselors and academic staff is key to ensuring consistent, integrated support for students as they prepare for meaningful and sustainable careers.

## Conclusion

This study examined the relationships between OFTP dimensions, perceptions of decent work, and employability among Chinese college students. The results show that focus on opportunities and perceived remaining time are significant positive correlates of employability, both directly and through their associations with perceptions of decent work. In contrast, focus on limitations has a weak negative association, underscoring the challenges of a constrained future outlook.

These findings highlight the importance of fostering future-oriented mindsets and positive expectations about equitable and meaningful work. For Chinese colleges, actionable strategies include integrating career-focused education into curricula and offering psychological support to reinforce students’ confidence and engagement with long-term career planning. By addressing both psychological and contextual influences, institutions can better prepare students for sustainable and meaningful participation in an increasingly competitive labor market.

## Data Availability

The raw data supporting the conclusions of this article will be made available by the authors, without undue reservation.

## References

[ref1] Al-Fattal A. (2025). Marketing challenges in entrepreneurship: perspectives from business students. Merits 5:7. doi: 10.3390/merits5010007

[ref2] Ayvaz A. Karacan-Özdemir N. (2024). The role of contextual predictors and psychosocial resources in the school-to-work transition. Career Dev. Q. 72, 346–365. doi: 10.1002/cdq.12365

[ref6] Cheung R. Jin Q. Cheung C.-k. (2018). Perceived employability of nonlocal Chinese university students in Hong Kong: the impact of acculturative and vocational variables. J. Career Assess. 26, 137–153. doi: 10.1177/1069072716680045

[ref7] Chu M. L. Creed P. A. Conlon E. G. (2021). Work–study boundary congruence, contextual supports, and proactivity in university students who work: a moderated-mediation model. J. Career Dev. 48, 166–181. doi: 10.1177/0894845319830253

[ref8] de Sousa C. Romana F. A. (2024). Training to achieve sustainable employment for youth and young adults. Merits 4, 118–131. doi: 10.3390/merits4020009

[ref9] Dinis A. C. Ferraro T. Pais L. dos Santos N. R. (2024). Decent work and burnout: A profile study with academic personnel. Psychol. Rep. 127, 335–364. doi: 10.1177/00332941221100454, 35603670 PMC10782657

[ref10] Duffy R. D. Blustein D. L. Diemer M. A. Autin K. L. (2016). The psychology of working theory. J. Couns. Psychol. 63, 127–148. doi: 10.1037/cou0000140, 26937788

[ref11] Duffy R. D. Gerdel S. Kim H. J. Choi Y. (2024). Experiencing work as decent, meaningful, neither, or both: a latent profile analysis. J. Posit. Psychol. 19, 686–698. doi: 10.1080/17439760.2023.2257631

[ref12] Duffy R. D. Prieto C. G. Kim H. J. Raque-Bogdan T. L. Duffy N. O. (2021). Decent work and physical health: a multi-wave investigation. J. Vocat. Behav. 127:103544. doi: 10.1016/j.jvb.2021.103544

[ref13] Fang C. Ran Y. Huan Z. Zhang X. (2025). The mediating role of decent work perception in role clarity and job embeddedness among Chinese nurses: A cross-sectional study. BMC Nurs. 24:225. doi: 10.1186/s12912-025-02861-z, 40016736 PMC11869399

[ref14] Fasbender U. Wöhrmann A. M. Wang M. Klehe U.-C. (2019). Is the future still open? The mediating role of occupational future time perspective in the effects of career adaptability and aging experience on late career planning. J. Vocat. Behav. 111, 24–38. doi: 10.1016/j.jvb.2018.10.006

[ref15] Ferraro T. Moreira J. M. dos Santos N. R. Pais L. Sedmak C. (2018a). Decent work, work motivation and psychological capital: an empirical research. Work 60, 339–354. doi: 10.3233/WOR-182732, 29966216

[ref16] Ferraro T. Pais L. Moreira J. M. Santos N. R. D. (2018b). Decent work and work motivation in knowledge workers: the mediating role of psychological capital. Appl. Res. Qual. Life 13, 501–523. doi: 10.1007/s11482-017-9539-2

[ref19] Gaweł A. Krstić Z. (2021). Gender gaps in entrepreneurship and education levels from the perspective of clusters of European countries. J. Dev. Entrep. 26, 1–22. doi: 10.1142/S1084946721500248

[ref22] Hair J. F. Hult G. T. M. Ringle C. M. Sarstedt M. (2019). A primer on partial least squares structural equation modeling (PLS-SEM). 2nd Edn. London: SAGE Publications.

[ref25] Ho H. C. Yeung D. Y. (2016). Effects of occupational future time perspective on managing stressful work situations. Int. J. Psychol. 51, 261–268. doi: 10.1002/ijop.12144, 25623057

[ref27] Janeiro I. N. Duarte A. M. Araújo A. M. Gomes A. I. (2017). Time perspective, approaches to learning, and academic achievement in secondary students. Learn. Individ. Differ. 55, 61–68. doi: 10.1016/j.lindif.2017.03.007

[ref31] Kock N. Lynn G. S. (2012). Lateral collinearity and misleading results in variance-based SEM: an illustration and recommendations. J. Assoc. Inf. Syst. 13, 546–580. doi: 10.17705/1jais.00302

[ref32] Lam C. C.-C. Cheung F. Wu A. M. (2019). Job insecurity, occupational future time perspective, and psychological distress among casino employees. J. Gambl. Stud. 35, 1177–1191. doi: 10.1007/s10899-019-09855-y, 31069612

[ref33] Ling H. Teng S. Liu X. Wu J. Gu X. (2022). Future work self salience and future time perspective as serial mediators between proactive personality and career adaptability. Front. Psychol. 13:824198. doi: 10.3389/fpsyg.2022.824198, 35572329 PMC9094421

[ref35] Ma Y. Huang G. Autin K. L. (2021). Linking decent work with academic engagement and satisfaction among first-generation college students: a psychology of working perspective. J. Career Assess. 29, 148–163. doi: 10.1177/1069072720943153

[ref37] Nam J. S. Kim S. Y. (2019). Decent work in South Korea: context, conceptualization, and assessment. J. Vocat. Behav. 115:103309. doi: 10.1016/j.jvb.2019.05.006

[ref38] Potts D. (2022). Employability development and career outcomes from short-term learning abroad programmes. High. Educ. Res. Dev. 41, 1215–1230. doi: 10.1080/07294360.2021.1901665

[ref39] Renz F. M. Vogel J. U. (2024). Be the change you want to see: problem-based learning to promote diversity, justice, equity, inclusion, belonging, and sustainability in the classroom and workplace. Merits 4, 79–94. doi: 10.3390/merits4010006

[ref40] Ringle C. M. Wende S. Becker J.-M. 2024. SmartPLS 4. In SmartPLS. Availabl eonline at: https://www.smartpls.com (Accessed January 15, 2025).

[ref42] Rothwell A. Herbert I. Rothwell F. (2008). Self-perceived employability: construction and initial validation of a scale for university students. J. Vocat. Behav. 73, 1–12. doi: 10.1016/j.jvb.2007.12.001

[ref43] Rothwell A. Jewell S. Hardie M. (2009). Self-perceived employability: investigating the responses of post-graduate students. J. Vocat. Behav. 75, 152–161. doi: 10.1016/j.jvb.2009.05.002

[ref45] Seol J. H. Sohn Y. W. Yoo M. Park Y. (2024). Decent work, posttraumatic stress disorder, and posttraumatic growth from the psychology of working perspective: A three-wave study of military personnel. J. Career Assess. 32, 26–47. doi: 10.1177/10690727231163321

[ref46] Shen J. Zhang B. Huang W. (2024). Attaining decent work among Chinese rural migrants: exploring the roles of psychological ownership and proactive personality within the psychology of working theory. J. Couns. Psychol. 71, 126–137. doi: 10.1037/cou0000713, 38300563

[ref48] Shiyuan Y. Jinxiu Y. Jingfei X. Yuling Z. Longhua Y. Houjian L. . (2022). Impact of human capital and social capital on employability of Chinese college students under COVID-19 epidemic—joint moderating effects of perception reduction of employment opportunities and future career clarity. Front. Psychol. 13:1046952. doi: 10.3389/fpsyg.2022.1046952, 36605287 PMC9809468

[ref49] Smith R. W. Baranik L. E. Duffy R. D. (2020). Psychological ownership within psychology of working theory: A three-wave study of gender and sexual minority employees. J. Vocat. Behav. 118:103374. doi: 10.1016/j.jvb.2019.103374

[ref50] Soares J. Taveira M. Silva A. D. (2024). Protean career orientation scale: A validation study with Portuguese university students. Psicologia 38, 14–24. doi: 10.17575/psicologia.1920

[ref52] Steindórsdóttir B. D. Sanders K. Nordmo M. Dysvik A. (2024). A cross-lagged study investigating the relationship between burnout and subjective career success from a lifespan developmental perspective. J. Occup. Organ. Psychol. 97, 273–300. doi: 10.1111/joop.12471

[ref54] Tian H. (2023). Exploration of the relationship between the quality of higher education and the employability of college students. J. Contemp. Educ. Res. 7, 78–84. doi: 10.26689/jcer.v7i4.4879

[ref55] Van der Heijden B. I. J. M. Le Blanc P. M. Hernandez A. Gonzalez-Roma V. Yeves J. Gamboa J. P. (2019). The importance of horizontal fit of university student jobs for future job quality. Career Dev. Int. 24, 239–256. doi: 10.1108/CDI-12-2018-0330

[ref56] Wan W. Duffy R. D. (2023). Decent work and turnover intentions among Chinese millennials: A longitudinal study. J. Career Dev. 50, 933–946. doi: 10.1177/08948453221133831

[ref58] Wang D. Guo D. Song C. Hao L. Qiao Z. (2022). General self-efficacy and employability among financially underprivileged Chinese college students: the mediating role of achievement motivation and career aspirations. Front. Psychol. 12:719771. doi: 10.3389/fpsyg.2021.719771, 35126222 PMC8815425

[ref59] Wang D. Jia Y. Hou Z.-J. Xu H. Zhang H. Guo X.-L. (2019). A test of psychology of working theory among Chinese urban workers: examining predictors and outcomes of decent work. J. Vocat. Behav. 115:103325. doi: 10.1016/j.jvb.2019.103325

[ref61] Wang Z. Yang L. Zhu Y. Tang X. Wang T. Chen L. . (2024). Innovative behavior and structural empowerment among the Chinese clinical nurses: the mediating role of decent work perception. BMC Nurs. 23:881. doi: 10.1186/s12912-024-02554-z, 39627823 PMC11613594

[ref62] Wang J. Ye Z. Chang B. (2024). The association between perceived social support and future decent work perception: A moderated mediation model. Acta Psychol. 249:104458. doi: 10.1016/j.actpsy.2024.104458, 39121615

[ref63] Wei J. Chan S. H. J. Autin K. (2022). Assessing perceived future decent work securement among Chinese impoverished college students. J. Career Assess. 30, 3–22. doi: 10.1177/10690727211005653

[ref64] Wen Y. Chen H. Liu F. Wei X. (2023). The relationship between career calling and resilience among rural-oriented pre-service teachers: the chain mediating role of career adaptability and decent work. Behav. Sci. 14:11. doi: 10.3390/bs14010011, 38247663 PMC10813303

[ref65] Wen G. Qing X. (2024). A research on mix-teaching method: characteristics of employment education teachers on employability of students in Chinese institutions. Psychol. Sch. 61, 1922–1943. doi: 10.1002/pits.23143

[ref66] Wong L. P. (2024). Artificial intelligence and job automation: challenges for secondary students’ career development and life planning. Merits 4, 370–399. doi: 10.3390/merits4040027

[ref67] Xu Y. Liu D. Tang D. S. (2022). Decent work and innovative work behaviour: mediating roles of work engagement, intrinsic motivation and job self-efficacy. Creat. Innov. Manag. 31, 49–63. doi: 10.1111/caim.12480

[ref68] Yan Y. Geng Y. Gao J. (2023). Measuring the decent work of knowledge workers: constructing and validating a new scale. Heliyon 9:e17945. doi: 10.1016/j.heliyon.2023.e17945, 37496922 PMC10366389

[ref70] Yeung D. Y. Ho A. K. K. (2020). Focus on opportunities or limitations? Their effects on older workers’ conflict management. Front. Psychol. 11:571874. doi: 10.3389/fpsyg.2020.571874, 33224065 PMC7674287

[ref71] Yizhong X. Lin Z. Baranchenko Y. Lau C. K. Yukhanaev A. Lu H. (2017). Employability and job search behavior: a six-wave longitudinal study of Chinese university graduates. Employee Relat. 39, 223–239. doi: 10.1108/ER-02-2016-0042

[ref72] Zacher H. (2013). Older job seekers' job search intensity: the interplay of proactive personality, age and occupational future time perspective. Ageing Soc. 33, 1139–1166. doi: 10.1017/S0144686X12000451

[ref73] Zacher H. Frese M. (2009). Remaining time and opportunities at work: relationships between age, work characteristics, and occupational future time perspective. Psychol. Aging 24, 487–493. doi: 10.1037/a0015425, 19485664

[ref75] Zhang X. Zhang L. Xue B. Li Y. Yan M. Luo H. . (2024). Effort–reward imbalance and well-being among psychiatric nurses: the mediating role of burnout and decent work. BMC Nurs. 23:635. doi: 10.1186/s12912-024-02301-4, 39256745 PMC11389592

