# Supplementary material for: Occupational future time perspective and employability: the role of decent work perceptions in Chinese college students
Source: Front Psychol. 2026 Jan 23;16:1712125. doi: 10.3389/fpsyg.2025.1712125 (PMC12875975; doi:10.3389/fpsyg.2025.1712125)
Supplement: Supplementary file 1 [file Data_Sheet_1.PDF]

Table S1. Outer loadings of the measurement model

Indicator	Decent Work	Employability	Focus limitations	Focus opportunities	Remaining time
DW1	0.709				
DW2	0.645				
DW3	0.706				
DW4	0.670				
DW5	0.784				
DW6	0.813				
DW7	0.730				
DW8	0.651				
DW9	0.735				
Employability1		0.825			
Employability2		0.897			
Employability3		0.849			
Employability4		0.828			
Employability5		0.780			
Employability6		0.800			
FL1			0.913		
FL2			0.904		
FL 3 Item 5 excluded			-0.345		
FO1				0.905	
FO2				0.811	
FO3				0.931	
FO4				0.841	
RT1					0.945
RT2					0.946

Note. Values represent standardized outer loadings of the indicators on their respective latent constructs. Only primary loadings are reported.

Table S2. Collinearity Statistics. VIF

Item	VIF
DW1	2.685
DW2	2.188
DW3	2.258
DW4	1.715
DW5	2.257
DW6	2.292
DW7	2.008
DW8	1.720
DW9	2.159
Employability1	2.410
Employability2	3.673
Employability3	2.715
Employability4	2.317
Employability5	1.980
Employability6	1.993
FL1	1.741
FL2	1.741
FO1	3.513
FO2	1.982
FO3	4.186
FO4	2.094
RT1	5.839
RT2	6.032
RT3	2.199
